# Supplementary material for: Root Colonization by Fungal Entomopathogen Systemically Primes Belowground Plant Defense against Cabbage Root Fly
Source: J Fungi (Basel). 2022 Sep 16;8(9):969. doi: 10.3390/jof8090969 (PMC9505207; doi:10.3390/jof8090969)
Supplement: Supplementary file 1 [file jof-08-00969-s001.zip › jof-1871063-supplementary.pdf]

Supplementary material

Article title

Root colonization by fungal entomopathogen systemically primes belowground plant defense against cabbage root fly

Authors

Catalina Posada-Vergara [1], Katharina Lohaus [1], Mohammad Alhussein [2], Stefan Vidal [1], and Michael Rostás [1]*.

[1] Agricultural Entomology, Department of Crop Sciences, University of Göttingen, Grisebachstr. 6, 37077 Göttingen, Germany; e-mail@e-mail.com

[2] Molecular Phytopathology and Mycotoxin Research, University of Göttingen, 37077 Goettingen, Germany

***** Correspondence: michael.rostas@uni-goettingen.de

Table S1. List of the fungal strains used in the study (see (Hettlage, 2018)).

Strain	Crop regime	Location
GC1I	Semi-natural permanent grassland for at least 7 years. Grassland harvested twice a year	51°34'10.7"N 10°03'54.1"E
Gd12		
Cb15III	Winter wheat, barley or oilseed rape crop rotation, conventional farming	51°33'58.3"N 10°04'10.1"E
Cb17b		
CC5		

Table S2. Primers used for qPCR amplification of genes of *Brassica napus* root tissues

ID	Gen/accession	Pathway	Gene description	Sequence 5' --> 3'	Reference
**ABA2**	LOC106300040<br>XM_013736089	Abscisic acid biosynthesis	Xanthoxin dehydrogenase	GCATCGCTCGTCTGTTCCAC<br>CGGCGAAGTCAACAGCGTTA	Karssemeijer et al., 2021
**ERF2**	At5g47220	Ethylene (ET) signaling	Ethylene Response Factor 2	ATGTACGGACAGAGCGAGGT<br>AAGCTTCGAAACCAACAAGTAACTG	Yang et al., 2010
**ACO**	EV102889	ET biosynthesis	ACC oxidase	TCCGTCTGGGCTATCACTCT<br>GTGAGTGGGTCGATGTTCCT	Maag 2014
**PR1**	XM_013877950	Salicylic acid (SA) signaling	Pathogenesis-related protein 1	AAAGCTACGCCGACCGACTACGAG<br>CCAGAAAAGTCGGCGCTACTCCA	Alkooranee 2017
**PAL**	LOC106342153<br>XM_013781008	SA synthesis-Phenylpropanoid pathway	phenylalanine ammonia-lyase 1	TCGCTATGGCTTCTTACTGCTCTG<br>GAGGTCTTACGAGATGAGATGAGTCC	Karssemeijer et al., 2021
**AOS**	LOC106327419<br>XM_013765565	Jasmonic acid (JA) synthesis	Allene oxide synthase	ACCGCTTGCGACTAGGGATC<br>CAAAGTCCTTACCGGCGCAC	Karssemeijer et al., 2021
**MYC2**	EV120351	JA signaling	Basic helix-loop-helix (bHLH) DNA-binding family protein	GCAAAGCCCAGACAGAGAAC<br>AGCTCACGCAACACCTTCTT	Maag et al., 2014
**TPI**	EV144353	JA signaling	Trypsin inhibitor B-like	GTGGTATCACCATGAACCTTG<br>GTTGACCACCTTAACCGGAA	Maag et al., 2014
**PDF1.2**	EV163328	JA signaling	Defensin-like protein 16	TCCATCACCCTTCTCTTTGC<br>TTTTGGCACGCATAGTCGTA	Maag et al., 2014
**ACTIN**	AF111812	Housekeeping gene	Housekeeping gene	ATCGTCCTCAGTGGTGGTTC<br>TTGATCTTCATGCTGCTTGG	Maag et al., 2014
**GTR1A2**	Bra018096	Glucosinolate (GSL) transport	Glucosinolate Transporter 1 A2	ATTCACCTTCGGGGAACTGG<br>TCGCTTGCTTCTGCTTGGTC	(Sontowski et al., 2019)
**CYP79B2**	At4G39950	Indole GSLs biosynthesis	CYTOCHROME P450, FAMILY 79, SUBFAMILY B, POLYPEPTIDE 2	AAGAGGTTGTGCTGCTCCG<br>TCCAAGTGAAACCTTGAAGAAGTC	Tytgat et al., 2013 Also in Marthur
**CYP83A1**	At4G13770	Aliphatic GSL biosynthesis	CYTOCHROME P450, FAMILY 83, SUBFAMILY A, POLYPEPTIDE 1	CTCCTTATCCCTCGTGCTTG<br>TGTCGTAACCAGCGATCTTG	Mathur et al., 2013
**BABG**	LOC106429220<br>XM_022718558	Myrosinase biosynthesis	Beta-glucosidase 27-like	CCGAGCGAGCTATGGAGTTT<br>CGGCTTGTCTGGATCCACTT	This study
**BnMyr4**	LOC106430598<br>XM_013871387	Myrosinase biosynthesis	PREDICTED myrosinase 4-like	TCAACTGCGACAATCCCCTT<br>ATCACAAGCAAGGTCTCCGG	This study
**Myr2.Bn1**	LOC106382545<br>NM_001316199	Myrosinase biosynthesis	B.napus myrosinase, thioglucoside glucohydrolase	TTGAAGGAGGGAGAGGTCGT<br>AGCATTGAGTTCGCCCATCA	This study
**DTCMT.a**	LOC106392535<br>XM_013833342	Phytoalexin brassinin biosynthesis	Dithiocarbamate S-Methyltransferase	TGTTCCACTGGACCTAACACG<br>GGCCAAAGAAAGATCCGGGA	This study

Table S3. Acquisition parameters for phytohormones analysis.

Compound	RT [min]	Polarity	Parent Ion [m/z]	Fragmentor V	Collision Energy V	Product Ion [m/z]
Trans-zeatin	1.93	+	220.1	100	15	136.1
					9	202.1
					22	148.1
Trans-zeatin-d5	1.92	+	225.1	105	16	137.1
					10	207.2
Abscisic acid (ABA)	4.68	-	263.1	85	4	153.1
					5	219.1
					12	204.1
Abscisic acid-d6	4.67	-	269.1	88	4	159.1
					8	225.1
Jasmonic acid (JA)	5.20	+	211.2	85	8	133.1
					8	151.1
					5	193
Jasmonic acid-d5	5.19	+	216.1	85	8	135.1
					9	153.2
					7	198.2
Salicylic acid (SA)	4.05	-	137	140	15	93.1
					35	65.1
Salicylic acid glucoside (SA-Glu)	2.41	-	137	140	15	93.1
					35	65.1

Table S4. Statistical summary of GLM analysis with binomial distribution of the total mortality on in vitro experiments. Mortality includes mycosed larvae, pupae, and adults that developed mycosis after emerging.

**Isolate**	**L3 Larvae**			
	Estimate	SEM	z value	Pr(>|z|)
Intercept	-1.6398	0.3706	-4.425	9.65E-06***
Cb15III	2.339	0.4349	5.378	7.52E-08***
Cb17B	3.0474	0.4525	6.735	1.64E-11***
CC5	3.1991	0.4588	6.972	3.11E-12***
Gc1I	4.1194	0.5248	7.85	4.15E-15***
Gd12	4.1194	0.5248	7.85	4.15E-15***
Sand	-0.7047	0.2525	-2.791	0.00525**

Table S5. Statistical summary of GLM analysis with binomial distribution of the total mortality in planta experiment. Mortality includes mycosed larvae, pupae, and adults that developed mycosis after emerging. RCD: root collar diameter, included as covariant.

Factor	Estimate	Std. Error	z value	Pr(>|z|)
(Intercept)	2.9015	0.9581	3.028	0.00246**
Cb15III	-0.8301	0.3658	-2.269	0.02324*
Cb17B	-0.2827	0.3721	-0.76	0.44746
CC5	-0.4439	0.368	-1.206	0.22774
Gc1I	-0.853	0.3887	-2.195	0.02818*
Gd12	-1.0107	0.3561	-2.839	0.00453**
RCD	-0.1567	0.111	-1.412	0.15794

Table S6. Statistical summary of beta regression analysis with of percentage of damage of root collar in planta experiment.

Factor	Estimate	Std. Error	z value	Pr(>|z|)
(Intercept)	1.1920	0.1789	6.662	2.71e-11 ***
TreatCb15III	-0.7290	0.2491	-2.926	0.003432 **
TreatCb17B	-0.8382	0.2379	-3.523	0.000427 ***
TreatCC5	-0.6718	0.2394	-2.807	0.005005 **
TreatGc1I	-0.8858	0.2623	-3.377	0.000732 ***
TreatGd12	-1.1614	0.2415	-4.808	1.52e-06 ***

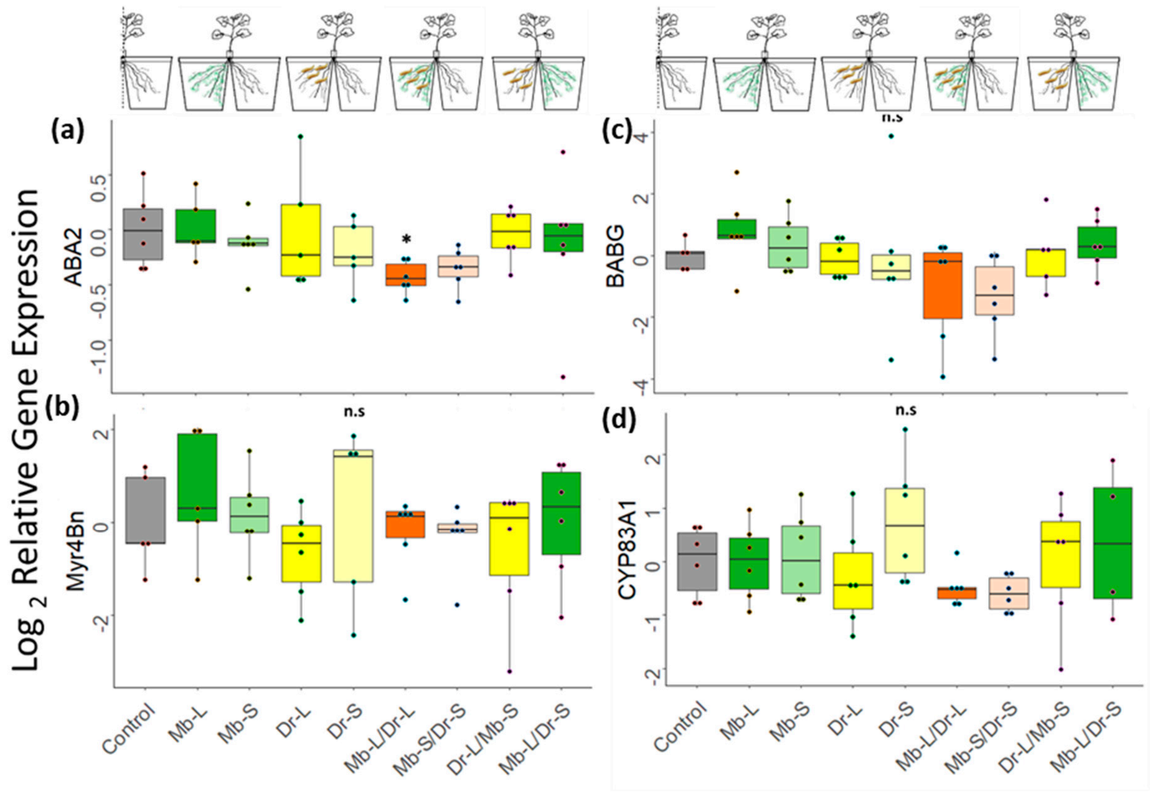

Figure S1. Normalized expression of representative genes of (**a**)abscisic acid biosynthesis *ABA2*, (**b**) myrosinase synthesis *Myr4Bn*, (**c**) beta-glucosidase biosynthesis *BABG* and (**d**) aliphatic GSL synthesis *CYP83A1*. Plants grew in a split root setup in which each compartment had either *M. brunneum* inoculation (Mb) in the local (L) or adjacent (S) compartment, *D. radicum* egg infestation (Dr) in the local (L) or adjacent (S) compartment, both treatments in same compartment (Mb-L/Dr-L), or each in adjacent compartments of the same plant (Dr-L/Mb-S; Mb-L/Dr-S). Eggs were placed 4 weeks after Mb inoculation. Plants were harvested 7 days after egg infestation. Gene expression was normalized to the housekeeping gene *AUXIN*. The boxplot shows all data points from at least 4 independent biological replicates (n ≤ 6) in which the horizontal line represents the median, surrounded by the upper (25th) and lower (75th) percentiles.
